# Structural and micro-anatomical changes in vertebrae associated with idiopathic-type spinal curvature in the *curveback *guppy model

**DOI:** 10.1186/1748-7161-5-10

**Published:** 2010-06-07

**Authors:** Kristen F Gorman, Gregory R Handrigan, Ge Jin, Rob Wallis, Felix Breden

**Affiliations:** 1 Department of Biological Sciences, Simon Fraser University, Burnaby, British Columbia, Canada; 2 Department of Oral Health Sciences, Faculty of Dentistry, Life Sciences Institute, University of British Columbia, Vancouver, British Columbia, Canada

## Abstract

**Background:**

The *curveback *lineage of guppy is characterized by heritable idiopathic-type spinal curvature that develops during growth. Prior work has revealed several important developmental similarities to the human idiopathic scoliosis (IS) syndrome. In this study we investigate structural and histological aspects of the vertebrae that are associated with spinal curvature in the *curveback *guppy and test for sexual dimorphism that might explain a female bias for severe curve magnitudes in the population.

**Methods:**

Vertebrae were studied from whole-mount skeletal specimens of curved and non-curved adult males and females. A series of ratios were used to characterize structural aspects of each vertebra. A three-way analysis of variance tested for effects of sex, curvature, vertebral position along the spine, and all 2-way interactions (*i.e*., sex and curvature, sex and vertebra position, and vertebra position and curvature). Histological analyses were used to characterize micro-architectural changes in affected vertebrae and the intervertebral region.

**Results:**

In *curveback*, vertebrae that are associated with curvature demonstrate asymmetric shape distortion, migration of the intervertebral ligament, and vertebral thickening on the concave side of curvature. There is sexual dimorphism among curved individuals such that for several vertebrae, females have more slender vertebrae than do males. Also, in the region of the spine where lordosis typically occurs, curved and non-curved females have a reduced width at the middle of their vertebrae, relative to males.

**Conclusions:**

Based on similarities to human spinal curvatures and to animals with induced curves, the concave-convex biases described in the guppy suggest that there is a mechanical component to curve pathogenesis in *curveback*. Because idiopathic-type curvature in *curveback *is primarily a sagittal deformity, it is structurally more similar to Scheuermann kyphosis than IS. Anatomical differences between teleosts and humans make direct biomechanical comparisons difficult. However, study of basic biological systems involved in idiopathic-type spinal curvature in *curveback *may provide insight into the relationship between a predisposing aetiology, growth, and biomechanics. Further work is needed to clarify whether observed sex differences in vertebral characteristics are related to the female bias for severe curves that is observed in the population.

## Background

The *curveback *lineage of the guppy, *Poecilia reticulata *is characterized by heritable idiopathic-type spinal curvature that progresses during growth in otherwise healthy individuals. C*urveback *is derived from a single cross between a curved male and an unrelated non-curved female, and consists of curved and non-curved individuals [1]. Curved individuals display deviation of the spine primarily in the sagittal plane of the body as an anterior lordosis and posterior kyphosis in the tail (non-rib associated vertebrae) [2]. In addition to having a strong genetic component, prior study of the *curveback *phenotype has demonstrated several developmental similarities to the human idiopathic-type scoliosis (IS) syndrome. These include: curve onset after birth (guppies give birth to live fry), curve stabilization at sexual maturity, phenotypic variability (for age of curve onset, curve magnitude, and the propensity for a curve to stabilize or progress to severity), and a greater likelihood for females to develop severe curvature [1]. These parallels suggest that *curveback *is a feasible animal model for exploring the biological systems involved in heritable idiopathic-type spinal curvature. However, better characterization of the *curveback *phenotype is necessary to understand how the model might be applied to specific research questions.

Characteristically, spinal curvature in *curveback *lacks congenital anomalies (*e.g*. failure of formation or segmentation), although distortion of vertebral shape at and around the apex has been noted [1]. In order to characterize vertebral shape distortion associated with curvature in *curveback*, we compare structural and micro-anatomical differences among the vertebrae of curved and non-curved adults. The results are then related to studies regarding human spinal curvatures as well as studies involving vertebral distortion in animals.

## Methods

The guppy *P. reticulata *is a small, live-bearing teleost fish that is native to the streams of northeast South America. The species has been a model organism for ecological, evolutionary, developmental, and genetic research since the 1920's. The *curveback *lineage is composed of curved and non-curved individuals derived from a laboratory population that has been maintained by inbreeding since 2003. The lineage consists of multiple inbred families, all propagated from a single curved male. Laboratory fish are kept under standardized conditions [1] in compliance with protocols approved by the Simon Fraser University Animal Care Facility and the Canadian Council on Animal Care.

**Figure 1:**
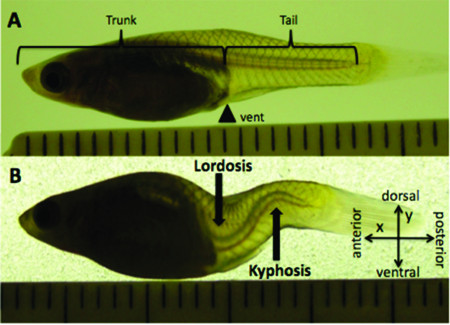
**The *curveback *phenotype**. **A**. Non-curved adult female from the *curveback *lineage, with trunk and tail portions of the body defined by being either anterior or posterior to the vent. **B**. Adult female with a qualitative curve score of 3 (Gorman, *et al.*, 2007). Scale shown in mm.

Offspring are born with a fully ossified skeleton after ~ 3 weeks of gestation, and sexual maturity is at about one month past birth. Curvature begins after birth (age of onset is variable) and stabilizes at around sexual maturity [1]. The phenotype analyzed for this study is a sagittal lordosis and kyphosis, both of variable magnitude, occurring in the tail (*i.e. *vertebrae posterior to the vent and not associated with ribs) (Figure 1). Males and females chosen for whole-mount skeletal analysis were matched for the magnitude of their curves based on qualitative scores defined in Gorman *et al*. (2007). All curves in this study were of a qualitative score 2 or 3, where 0 represents non-curved and 4 represents the greatest magnitude. Animals with curvature in the coronal plane were omitted from this study in order to make clear comparisons between vertebrae using photographs.

### Sample preparation

#### Whole-mount skeletal staining

Twenty-seven adult fish (14 females: 6 curved, 8 non-curved; 13 males: 6 curved, 7 non-curved) were stained for bone and cartilage. All fish were euthanized at a minimum of 3 months past birth and then fixed in 10% neutral-buffered formalin (Sigma). Specimens were fixed for 2 days, and then washed in several changes of distilled, deionized water (ddH_2_O) (>1 hour each), then dehydrated in 50% ethanol (EtOH) (24 h) and then 100% EtOH (24 h). Whole fish were then incubated in cartilage staining solution (8GX Alcian blue in 70% anhydrous EtOH and 30% acetic acid) for 24 hours at room temperature with mild agitation. Stained specimens were neutralized in saturated aqueous sodium borate solution for 9-12 hours and then bleached for 20 minutes (3% hydrogen peroxide and 1% potassium hydroxide). Specimens were then digested with a trypsin solution (1 g trypsin in 35 ml saturated sodium borate/65 ml ddH_2_O) until they were 60% clear. Bones were stained with Alizarin red solution in 0.1% potassium hydroxide solution at room temperature with mild agitation, until they were light red in colour. Specimens were destained in trypsin for 40-48 hours and then preserved in stepwise solutions of 30%glycerol/70% of 1% KOH, 60% glycerol, and 100% glycerol. Thymol crystals were added to glycerol to prevent fungal growth during long-term storage of specimens.

Whole-mount stained specimens were positioned on their side on a glass slide and viewed through a Meiji MEI (Tokyo, Japan) stereoscope under 4× and 2× magnification. Photographs were taken with a Pentax MX camera though a MA150/T2 (Meiji) adapter at 2× magnification. All photographs were taken so that the camera looked down on the profile of the fish. Measurements were taken from digitized photos (ImageJ software, http://rsbweb.nih.gov/ij/). Statistical analysis was conducted using JMP software for Mac, Version 7.0 (SAS Institute, INC., Cary, NC, USA).

#### Histological Analysis

Tails from 4 curved and 2 non-curved female *curveback *guppies were fixed overnight in 10% neutral-buffered formalin and then demineralized in a solution of 10% formaldehyde: 5% ethylenediaminetetraacetic acid (EDTA) for a minimum of 2 weeks. For half of the *curveback *specimens, tail vertebrae 4 and 11, which occur at the apex of the lordosis and kyphosis, respectively, were carefully dissected under a stereomicroscope. This was to ensure that they could later be precisely embedded for sectioning in the transverse plane. Tails to be sectioned in the sagittal plane were left intact.

Following several rinses in 1× phosphate-buffered saline (PBS), body segments were dehydrated through an ethanol: PBS series, cleared in xylene, and penetrated with paraffin under vacuum. Specimens were embedded under a stereomicroscope and sectioned in either the transverse or sagittal planes at a thickness of 7-10 μm. Optimal sectioning generally required that the blocks were first 'softened' by soaking in distilled water.

Tissue sections were stained with either Mallory's trichrome (as per [3]) or double-stained with Picrosirius Red and Alcian Blue (as per [4]). By the former protocol, collagen fibrils are stained blue. By the latter, collagen type I and bone appear red and sulfated proteoglycans, which are enriched in cartilage, as blue. Sections were photographed using a Hitachi HV-F22 3-CCD digital camera coupled to a Zeiss Axioskop compound microscope.

### Vertebral Shape Analysis

**Figure 2:**
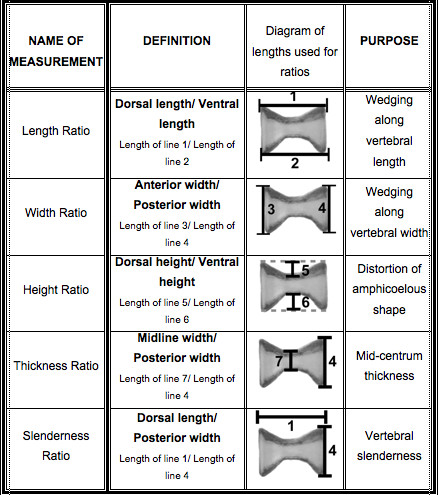
**Ratios used to characterize vertebral distortion associated with curvature**. To describe vertebral shape changes (*e.g*. asymmetry) independent of individual size differences, we calculated 5 ratios from 7 vertebral measurements.

Because curvature is manifested only in the tail, our analysis is limited to the vertebrae therein. As with humans, the length of a vertebra is described as parallel to the cranial-caudal axis, and the width is perpendicular. To describe vertebral shape changes (*e.g*. asymmetry) independent of individual size differences, we calculated 5 ratios from 7 vertebral measurements (Figure 2). The ratio of vertebral height to width was used as an index of vertebral slenderness as per several human studies [5-9]; vertebral thickness refers to the ratio of the width of the centrum at its flared end to the width at the constricted middle (Figure 2). All measurements were made by the same person and instrument in order to increase the precision of measurements and reduce variability introduced by measurement error.

For each ratio, individual effects from sex, curvature, position of vertebra in tail, and their interactions (sex*vertebra position, sex*curvature, vertebra position*curvature) were tested using a fixed-effects model ANOVA (three-way analysis of variance) (significance threshold: α = 0.05). Due to the fact the sample size for this study is small, we do not expect these tests to have statistical power sufficient for conclusive determinants of shape changes. Rather, our heuristic analysis uses ANOVA to compare means relative to variability within groups in order to detect general trends in vertebral shape.

For all ratios, vertebra position had a considerable effect. In order to identify which vertebrae were significantly contributing to these results, we separated the effects of vertebra position by conducting separate ANOVAs on each vertebra. Ratios with significant effects for either sex or curvature, but no sex*curvature interaction, were further investigated using one-way ANOVAs for either sex or curvature on data for each individual vertebra. Ratios with a significant interaction effect of sex *and *curvature were further investigated using a two-way ANOVA on data for each vertebra. For all data, the distribution of values was tested for normality using Shapiro-Wilks goodness-of-fit (α = 0.05). Statistical analyses were conducted using JMP software for Mac, Version 7.0 (SAS Institute, INC., Cary, NC, USA).

## Results

### The axial skeleton of *P. reticulata*

**Figure 3:**
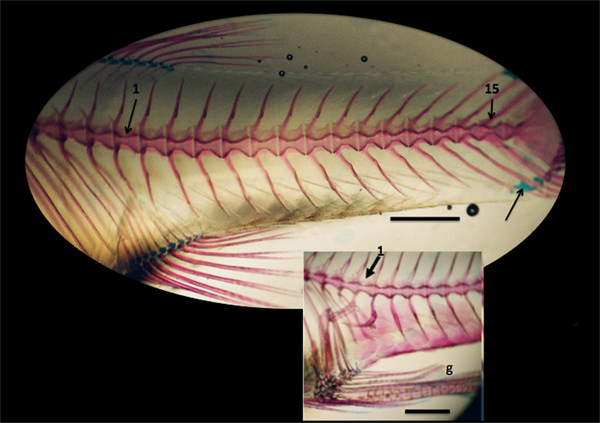
**The tail skeleton of the guppy *P. reticulata***. The first (1) and last (15) tail vertebrae, shown in an adult female. Cartilage that fixes the last three tail vertebrae to the caudal complex is stained with Alcian blue (arrow). Insert shows the haemal spines of first two vertebrae of an adult male that are transformed into the suspensorium for the gonopodium (g). Scale bar is 1 mm.

All curved and non-curved whole-mount specimens of *curveback *have 15 vertebrae in their tail. The first tail vertebra is identified as the anterior-most vertebra that is not associated with ribs (Figure 3). With the exception of the first two tail vertebrae, the vertebral column of the tail is a metameric structure with each vertebra comprising an amphicoelous (hour-glass shape) centrum, with paired neural arches on its dorsal face and haemal arches ventrally. Among the family Poeciliidae, the anal fin and vertebrae involved in its suspension are sexually dimorphic, so that in mature males, the anal fin is transformed into the gonopodium (an intromittent organ) and the first two tail vertebrae form its suspensorium (see Figure 3) [10]. The last three vertebrae in the tail (numbers 13, 14, 15) are fixed to the caudal complex by cartilage (apparent in Figure 3).

### Gross morphological changes in vertebrae associated with spinal curvature in *curveback*

**Figure 4:**
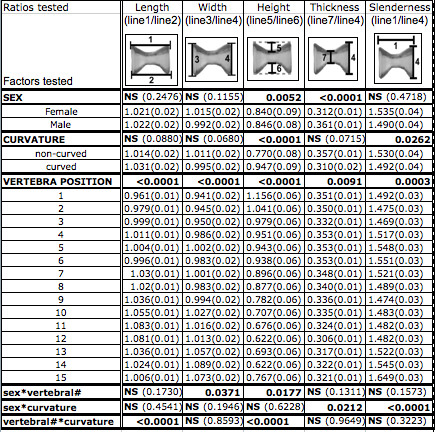
**Results from three-way analysis of variance for all 5 ratios**. For each ratio, individual effects from sex, curvature, vertebra position, and their interactions (sex*vertebra position, sex*curvature, vertebra position*curvature) were tested using a fixed-effects model ANOVA (three-way analysis of variance) (significance threshold: α = 0.05). Results for each factor tested (sex, curvature, position of the vertebra in the tail) and their interactions are shown in bold. The mean values for each component of a test factor are shown below the relevant test factor, with values for standard error in parenthesises. Standard error was calculated using a pooled estimate of error variance.

In order to detect asymmetry in the shape of vertebrae, 5 ratios were calculated from 7 measurements (Figure 2) of each of the 15 tail vertebrae. The distribution of data for all ratios was normal. Therefore, values for these ratios were analyzed using a three-way ANOVA that tested for the effects of sex, curvature, and vertebra position, as well as two-way interactions among individual factors. These results are summarized in Figure 4. Vertebral position along the tail had a significant effect on all ratios. The height and thickness ratios showed significant sexual dimorphism among all adults, and the slenderness ratio showed significant sexual dimorphism only among curved individuals. The effect of curvature was significant in height and slenderness ratios only, although there was a significant interaction from sex*curvature for the thickness ratio. The ratios with significant interactions from either sex*vertebral position (width ratio) or curvature*vertebral position (length ratio) were further investigated using a one-way ANOVA on data for each individual vertebra (Table 1). Because the thickness and slenderness ratios had a significant sex*curvature interaction, they were further investigated using a two-way ANOVA on data for each vertebra (Table 2).

**Table 1:** Vertebra specific one-way analysis of variance for length ratio and width ratios.

VERTEBRAL POSITION	LENGTH RATIO			WIDTH RATIO		
	**non-curved**	**curved**	**significance**	**female**	**male**	**significance**

1	0.984(0.02)	0.933(0.02)	0.105	0.917(0.02)	0.968(0.02)	0.114
2	0.992(0.02)	0.964(0.02)	0.243	0.937(0.02)	0.956(0.02)	0.580
3	1.007(0.02)	0.988(0.02)	0.459	0.974(0.03)	0.924(0.02)	0.169
4	1.015(0.02)	1.007(0.02)	0.705	1.016(0.02)	0.954(0.02)	0.057
5	1.012(0.02)	0.994(0.02)	0.486	1.021(0.02)	0.982(0.02)	0.160
6	1.023(0.02)	0.963(0.02)	**0.0118**	0.978(0.02)	0.989(0.02)	0.694
7	1.031(0.01)	1.028(0.02)	0.891	1.026(0.02)	0.974(0.02)	**0.0390**
8	1.010(0.02)	1.033(0.02)	0.379	0.994(0.02)	0.971(0.02)	0.332
9	1.021(0.02)	1.055(0.02)	0.238	0.999(0.02)	0.988(0.02)	0.709
10	1.011(0.02)	1.110(0.03)	**0.0100**	1.064(0.02)	0.988(0.02)	**0.0180**
11	1.026(0.03)	1.153(0.03)	**0.0021**	1.045(0.02)	0.984(0.03)	0.088
12	1.039(0.04)	1.135(0.04)	0.080	1.043(0.03)	0.982(0.02)	0.074
13	1.029(0.01)	1.044(0.02)	0.446	1.083(0.03)	1.029(0.03)	0.146
14	1.004(0.02)	1.048(0.02)	0.085	1.094(0.03)	1.084(0.03)	0.822
15	1.002(0.02)	1.010(0.02)	0.743	1.042(0.03)	1.107(0.03)	0.138

**Length ratio: **because there was a significant interaction between vertebra position (*i.e*. number in tail) and curvature (see Figure 4), we further compared length ratios among individual vertebrae for curved and non-curved individuals. **Width ratio: **because there was a significant interaction between sex and vertebra position (see Figure 4), we further investigated the width ratio among individual vertebrae for males and females. Comparisons conducted with one-way ANOVA, (significance threshold: α = 0.05). Significant results are shown in bold. Mean values for each vertebra is shown with the standard error in parenthesises. Standard error was calculated using a pooled estimate of error variance.

**Table 2:** Vertebra specific two-way analysis of variance for height, thickness, and slenderness ratios

VERTEBRAL POSITION	HEIGHT RATIO			THICKNESS RATIO			SLENDERNESS RATIO		
	**sex**	**curve**	sex*curve	**sex**	**curve**	sex*curve	**sex**	**curve**	sex*curve

1	0.087	** < 0.0001**	0.140	** < 0.0001**	**0.0419**	0.137	0.346	**0.0139**	**0.0202**
2	0.066	**0.0002**	0.301	**0.0003**	.0690	0.071	0.076	**0.0482**	0.161
3	0.398	**0.0059**	0.863	**0.0014**	0.183	0.142	**0.0018**	0.508	**0.0426**
4	0.495	**0.0004**	0.669	**0.0132**	**0.0078**	0.383	**0.0053**	0.260	**0.0202**
5	0.963	**0.0004**	0.489	**0.0021**	**0.0035**	0.330	**0.0002**	0.565	**0.0160**
6	0.460	**0.0390**	0.702	**0.0280**	0.185	0.737	0.457	0.965	**0.0156**
7	0.761	**0.0038**	0.948	**0.0186**	0.078	0.719	0.302	0.319	**0.0038**
8	0.734	**0.0141**	0.730	0.078	**0.0250**	0.576	0.781	0.401	0.632
9	0.151	0.750	0.645	**0.0230**	**0.0041**	0.976	0.095	0.199	0.140
10	0.299	**0.0261**	0.893	0.270	**0.0045**	0.713	0.567	0.792	0.280
11	**0.0432**	**0.0002**	0.058	0.137	**0.0163**	0.575	0.743	0.330	0.290
12	0.066	**0.0002**	0.076	0.272	0.058	0.477	0.150	0.533	0.595
13	0.312	**0.0015**	0.708	0.291	**0.0265**	0.995	0.130	0.150	0.099
14	**0.0393**	**0.0482**	0.689	0.217	0.212	0.984	0.746	0.808	0.782
15	0.497	0.873	0.260	0.104	**0.0309**	0.857	0.877	0.233	0.866

Significant interactions (sex*vertebral position and vertebral position*curvature) for the height ratio (Figure 4) motivated us to investigate each vertebra using a two-way ANOVA. Also, because the thickness and slenderness ratios had a significant sex*curvature interaction, they were further investigated using a two-way ANOVA on data for each vertebra. Significant results are shown in bold, (significance threshold: α = 0.05).

**Figure 5:**
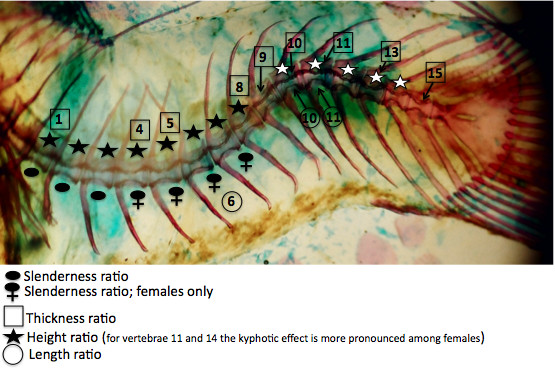
**Vertebrae significantly affected by curvature**. This representative whole mount skeleton shows the vertebrae that are most affected by curvature, as shown by analysis of ratios. In the region of lordosis, the amphicoelous centra of vertebrae 1-8 are distorted such that the height is increased on the dorsal side (black stars) and reduced on the ventral side, vertebra 6 is wedged such that the dorsal length is reduced relative to the ventral, and vertebrae 1,4,5,8 have reduced midline width. Curved females have more slender vertebra numbers 4,5,6,7 compared to curved males. In the region of kyphosis, vertebrae 10-14 have an increased height on the ventral side and reduced on the dorsal side (white stars), vertebrae 10 and 11 are wedged (have a reduced ventral length relative to their dorsal length), and vertebrae 9,10, 11, 13, 15 have reduced midline widths.

Because there was a significant interaction between vertebra position and curvature (p < 0.0001) for the length ratio, we further compared length ratios among individual vertebrae for curved and non-curved individuals. The results reveal that the mean length ratios for vertebrae 6 are significantly less in curved fish compared to non-curved (0.96, +/- 0.01 S.E. compared to 1.02, +/- 0.01 S.E.). Conversely, for vertebrae 10 and 11 the means are significantly higher in curved fish (vertebra 10: 1.11 +/- 0.02 S.E. compared to 1.01, +/- 0.02 S.E.; vertebra 11: 1.15, +/- 0.03 S.E. compared to 1.02, +/- 0.03 S.E. for non-curved). A normal amphicoelous vertebra is expected to have a length ratio of one; therefore to check whether the differences in curved vertebrae numbers 6, 10 and 11 are indicative of vertebral wedging, we tested whether the mean values from all vertebrae are significantly different from one (t-test, α = 0.05). Among non-curved fish the ratios are not significantly different from one, while among the curved fish, the ratios for vertebrae 6, 10 and 11 do significantly deviate from one. This indicates that vertebra 6 is slightly wedged such that the dorsal length is reduced relative to the ventral, and vertebrae 10 and 11 have a reduced ventral length relative to their dorsal length. These reduced lengths correspond to the concave side of curvature in lordosis and kyphosis (Table 1 and Figure 5).

Because there was a significant interaction between sex and vertebra position (p = 0.0371) for the width ratio, we further investigated the width ratio among individual vertebrae for males and females. The results show that the mean values for vertebrae 7 and 10 are significantly higher in females compared to males (Table 1- vertebra 7: 1.03+/-0.02 S.E. compared to 0.97, +/- 0.02 S.E., respectively; vertebra 10: 1.06, 0.02SE to 0.99, 0.02SE). To determine whether the vertebrae are actually wedged, we tested whether the mean values for curved males and females are significantly different from one (t-test, α = 0.05), which they were not.

The height ratio reflects the greatest change in vertebral shape that is associated with curvature, with significant effects from sex and curvature. Significant interactions (sex*vertebral position and vertebral position*curvature) motivated us to investigate each vertebra using a two-way ANOVA (Table 2). Among curved males and females, the normally amphicoelous shape of tail vertebrae 1-8 is distorted such that the height is increased on the dorsal side and reduced on the ventral side. For vertebrae 10-14 the height is increased on the ventral side and reduced on the dorsal side. The increase in dorsal height corresponds with lordosis, while the increase in ventral height with kyphosis. For vertebrae 11 and 14, this kyphotic distortion is more severe among females. Vertebrae 9 and 15 do not show significant distortion of the amphicoelous shape (Table 2, Figure 5).

The thickness ratio varied significantly with sex, and the slenderness ratio with curvature, and both ratios had a significant sex*curvature interaction (Figure 4). Therefore, these ratios were further investigated using a two-way ANOVA on data for each vertebra. The thickness ratio describes the width of the middle of the vertebral centrum, relative to the posterior width. Among non-curved and curved individuals, the vertebral thickness is sexually dimorphic such that among females the midline width is reduced for vertebrae 1-9. Among curved males and females, the midline width is reduced for vertebrae 1, 4, 5, 8-11, 13, 15 (Figure 5). The slenderness ratio describes the length of a vertebra relative to its width. Among curved individuals, the first three tail vertebrae have a reduced length relative to their width (compared to non-curved individuals). For vertebrae 4-7, among curved individuals, values for the slenderness ratio were sexually dimorphic such that vertebrae were more slender among females (Table 2, Figure 5).

### Micro-anatomical changes in lordotic and kyphotic *curveback *vertebrae

**Figure 6:**
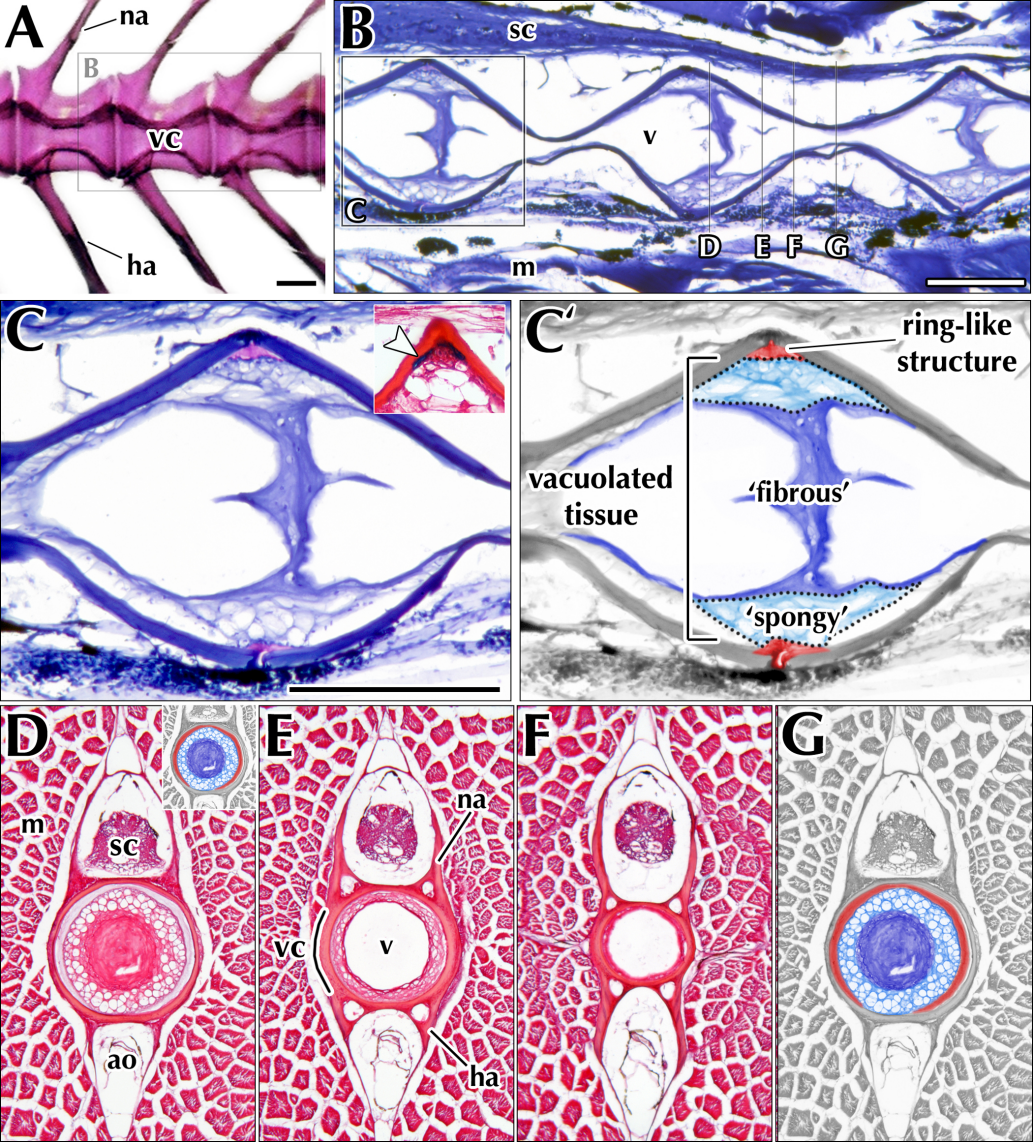
**Normal vertebral and intervertebral micro-anatomy of the guppy *P. reticulata***. (A) Whole-mount skeletal preparation of tail vertebrae shown in lateral view. Vertebral centra in *P. reticulata *are amphicoelous and bear paired arches dorsally and ventrally. (B-G) Micrographs of guppy vertebral sections stained by either Mallory's trichrome for collagen (B,C) or with a combination of Picrosirius Red and Alcian Blue (D-G), which stain for collagen and sulfated proteoglycans, respectively. (B) Sagittal section through vertebral column, showing three successive intervertebral ligaments (visible in whole-mount in boxed area in panel A). (C) Magnified view of boxed area in B. The intervertebral ligament of *P. reticulata *comprises two structures: an outer ring-like structure and a spongy, notochord-derived tissue that forms the core of the ligament and runs between adjacent centra. For clarity, these structures have been digitally colorized in panel C'. The spongy layer stains strongly for collagen, while the outer ring-like structure is enriched for sulfated proteoglycans (arrowhead in inset). (D-G) Consecutive transverse sections through a single vertebra, spanning the intervertebral region to the tapered middle of the centrum (see vertical lines in panel B for approximate axial level). Remnants of the embryonic notochord line the inside of the vertebral centrum and, at intervertebral levels, form a wall between vertebrae. Inset in panel D depicts a schematized intervertebral ligament in transverse section; colors correspond to those shown in panel C'. Abbreviations: ao, aorta; ha, hemal arch, m, axial muscle; na, neural arch; sc, spinal cord, v, vacuole; vc, vertebral centrum. Scale bar equals 200 μm.

Histological examination reveals that amphicoelous vertebral centra are coupled by non-cartilaginous intervertebral ligaments (Figure 6). Vertebral centra consist of acellular or osteoid bone, which lacks occluded osteocytes and is enriched with types I and III collagen, as revealed by staining with Picrosirius Red and Mallory's trichrome (Figure 6B-G). Vertebrae were negative for Alcian Blue staining (Figure 6D-G), indicating that they lack sulphated proteoglycans, a primary constituent of cartilage. This is consistent with most other advanced teleost species, which form vertebral body bone without a cartilage intermediate [11-17]. Vertebral centra encompass the remnants of the embryonic notochord [11,12,18,19]. This structure is highly vacuolated, particularly at the middle of the vertebral body, where it forms a single, great lumen enclosed by a thin outer sheath of fibrous tissue. At intervertebral levels, however, the notochord is considerably less vacuolated and more fibrous in composition. The guppy intervertebral ligament is built from the notochord and a ring-like structure that is fibrous in appearance and enriched for sulphated proteoglycans. For discussion, we subdivide the vacuolated inner core of the guppy intervertebral ligament into an outer 'spongy' layer and an inner 'fibrous' layer (Figure 6C).

**Figure 7:**
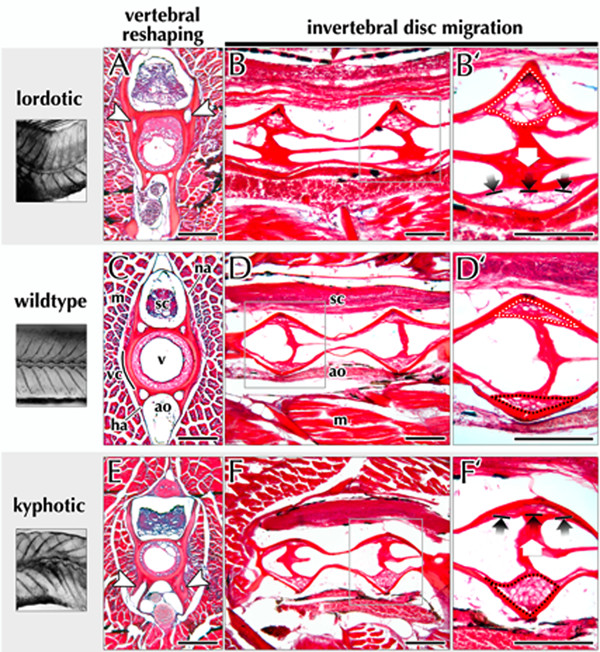
**Vertebral and intervertebral defects associated with spinal curvature in the *curveback *guppy**. Histological sections through normal and scoliotic vertebrae are presented for side-by-side comparison. Sections were cut in both the transverse and sagittal planes and stained with a combination of Picrosirius Red and Alcian Blue, which label collagen and sulfated proteoglycans, respectively. (A,C,E) Compared to normal vertebrae, lordotic and kyphotic vertebrae appear thicker and asymmetric in shape (white arrowheads) on the concave face of the spinal curve. This suggests that scoliotic vertebrae are being distorted or actively remodelled in response to biomechanical stress associated with curvature. (B,D,F) Within the intervertebral ligament of both lordotic and kyphotic vertebrae, the fibrous portion of the spongy layer (*cf*. Figure 6) appears to migrate towards the convex face of the spinal curvature. The white arrow in panel B' and F' indicates the direction of this migration. The displacement of the fibrous portion causes compression of the spongy portion on the convex face and distension on the opposite side (dotted outlines). Abbreviations: ao, aorta; ha, hemal arch; m, axial muscle; na, neural arch; v, vacuole; vc, vertebral centrum; sc, spinal cord. Scale bar equals 250 μm.

Micro-anatomical defects in both vertebral morphology and intervertebral orientation were observed in curved guppies (Figure 7A, 7E) as compared to non-curved (Figure 7B). Shape distortion was noted primarily on the side of the vertebra facing the concavity of the spinal curvature. Thus, lordotic vertebrae primarily display defects dorsally (Figure 7A) and kyphotic vertebrae are affected on their ventral surface (Figure 7E). In the transverse plane of section, the vertebral centra of scoliotic vertebrae appear slightly thicker; vertebral arches are more visibly affected, appearing either bilaterally asymmetric or else broadened at their base (Figure 7A, 7E). In the longitudinal plane, scoliotic vertebral centra appear bent in shape (Figure 7B, 7F).

The most pronounced defects associated with idiopathic-type curvature in the *curveback *guppy are in intervertebral ligaments occurring at the approximate apex of curvature (Figure 7B, 7F). For these ligaments, the fibrous layer appears to migrate towards the convex face of the spinal curvature in response to compressive forces acting on the concave side of the ligament (Figure 7B', 7F'). This displacement of the inner fibrous layer causes compression of the outer spongy layer on the convex face and distension on the opposite side. Thus, in intervertebral discs occurring at the apex of a lordotic curve, the inner fibrous layer migrates ventrally, while the spongy outer layer becomes distended dorsally (Figure 7B, 7B'). Conversely, in kyphotic curves, the fibrous layer is displaced dorsally and the spongy outer layer distended ventrally (Figure 7F, 7F').

## Discussion

### Structural and micro-anatomical changes associated with curvature in the *curveback *guppy

With lordosis, the concave side of the curve is dorsally directed, and with kyphosis the concavity is ventrally directed. Generally, we found that morphological change associated with lordosis and kyphosis is suggestive of anterior-posterior (*i.e*. cranial-caudal) compression along the spine. Structural evidence for this is from whole mount staining of skeletons showing that the normal amphicoelous (hour-glass) shape of vertebrae is distorted so that vertebral height is reduced on the convex and is greater on the concave side of curvature. In addition, vertebrae at the approximate apex of curvature (lordotic vertebra number 6 and kyphotic vertebrae 10 and 11) are wedged so that the length on the concave side of the curve is reduced relative to the convex length. Also, the midline width is significantly reduced for some vertebrae. Histological evidence for cranial-caudal compression is from micro-anatomical changes in both vertebrae and intervertebral ligaments (Figure 7). In curved guppies the centra and vertebral arch bases appear thicker on the side of vertebrae facing the concavity of a curve, and the spongy layer of the intervertebral ligament is displaced towards the convexity of the curve (Figure 7B, 7F), The observed changes in vertebral bone structure may be due to either (1) distortion of normal vertebral shape or (2) active remodelling of vertebral osteoid bone as a consequence of extrinsic forces. The latter phenomenon has been described in animal models with induced curvature [20-22], in the facets of patients with IS [23,24], as well as in a number of teleost species [12,14,25-28]. Further study in *curveback *should test whether vertebrae are undergoing active osteoid remodelling by volumetric analysis such as that employed by Kranenbarg and others [14,28].

There are similar concave-convex biases that occur along the cranial-caudal axis in the *curveback *and human and induced mammalian model curve phenotypes such as vertebral shape distortion, thickening of the bone, and intervertebral disc migration. With human idiopathic-type curvatures such as IS and Scheuermann kyphosis, as well as with curves associated with syndromes such as cerebral palsy, 3-M syndrome, and achondroplasia there is abnormal vertebral wedging (defined by a reduction in vertebral length on the concave side of a curve, with no significant length changes on the convex side) at and around the apex of curvature [29-36]. Vertebral wedging in human syndromes is often explained by the Hueter-Volkmann principle, which states that vertebral body growth is retarded by continuous or excessive mechanical compression upon the epiphysis (and stimulated by reduced compression) [37,38]. Distortion of vertebral shape consistent with the Hueter-Volkmann principle has been induced in animal models by direct mechanical stress (dog [39], monkey [40], calf tail [41], rat-tail [42,43], goat [44-46], rabbit [47], rat and rabbit and calf [38]). With induced models, asymmetrically loaded vertebrae demonstrate changes in growth rate along their growth plates, causing uneven progression in longitudinal growth and consequential shape distortion in the form of wedging [42]. In contrast to other animal models, the guppy has vertebrae that are composed of acellular bone (*i.e*. devoid of embedded osteocytes and formed by intramembranous ossification) (reviewed in [17]). Therefore further study of vertebral wedging in *curveback *should test cellular activity at the intervertebral region, the presumed growth zone of guppy vertebrae [16], to assess whether there is modulation of growth in curved individuals.

Recently, Driscoll and others [48] investigated the health of the intervertebral disc, migration of the nucleus pulposus, and trabecular bone mineral density (BMD) as indicators of concave-convex stresses associated with curvature in IS. In a finite element model, they demonstrated that increased BMD, annular degeneration, and disc migration played a moderate role in curve progression because they altered the path of force transmission within the spine. Considering that in *curveback*, there are concave-convex biases similar to those associated with IS [*i.e*. vertebral shape distortion, thicker bone on the concave side of curvature, displacement of the spongy layer of the intervertebral ligament (similar to the mammalian nucleus pulposus) toward the convexity of curvature] they may also contribute to curve progression in the guppy by altering the path of force transmission.

### Sexual dimorphism associated with spinal curvature

In human studies, the ratio of vertebral height/width has been used as an index of spinal slenderness, and females are reported to have more slender vertebrae than boys [5-9]. It has been suggested that sexual dimorphism for vertebral slenderness might be related to the greater likelihood for females with IS to develop curves of higher magnitude, because slenderness would reduce vertebral column stability and thus increase the susceptibility for a spine to buckle [8]. As in humans, the guppy curve phenotype is suggestive of column buckling, and the guppy vertebral column is subject to cranial-caudal loading (see [49]). Because in *curveback *there is a female bias for severe curves, we also compared the relative slenderness of vertebrae among males and females from the *curveback *lineage.

Among curved guppies, females tend to have more slender vertebrae in what appears to be the primary curve (lordosis). Vertebrae numbers 4-7 are more slender among curved females compared to non-curved males, non-curved females, and curved males (length of vertebrae relative to width). These results are similar to that described by Skogland and Miller (1981), who suggest that only a part of the vertebral column is more slender in girls with IS. It is important to note however, that investigations into the relationship between vertebral body slenderness, gender, and IS have given inconsistent results due to differences in sample size and constitution (*e.g.*, age), as well as whether one or multiple vertebrae were measured (see: [6-8,50]. Therefore, whether sexual dimorphism for vertebral slenderness is related to the observed female bias for severe curves in idiopathic-type scoliosis in humans is unclear.

We also found that among non-curved females of the *curveback *lineage, the first nine tail vertebrae have a relatively thinner midline width compared to non-curved males. A thicker midline width among non-curved males might be related to physiological and biomechanical factors associated with the male gonopodium and its suspensorium. Among the family Poeciliidae, the anal fin and vertebrae involved in its suspension (tail vertebrae 1 and 2) are sexually dimorphic, so that in mature males, the anal fin is transformed into the gonopodium (an intromittent organ) and the first two tail vertebrae form its suspensorium (see Figure 3) [11]. Among guppies, the length of the gonopodium can reach 50% of the total body length. In addition, as a part of courtship activities, the male guppy engages in extensive flicking, and swinging back and forth of the gonopodium. These activities have the potential to put sex-specific biomechanical strain on the supporting skeletal structure that could increase midline thickness. Therefore, further study is necessary to determine whether vertebral thinness is a predisposing risk factor for a greater curve magnitude among *curveback *females. Future studies should also examine the vertebrae of individuals who are not from the *curveback *lineage (*i.e. *lineages that do not exhibit spinal curvature).

### *Curveback *and its relation to spinal curvatures in humans

The *curveback *phenotype is primarily a sagittal plane deformity and so although previous studies have identified developmental parallels to IS [1,51], structurally the phenotype appears to be more similar to Scheuermann kyphosis. A limitation in our capacity to discuss comparisons between *curveback *and Scheuermann kyphosis is the lack of available studies regarding its natural history (reviewed in [29]). As a consequence, much of our comparisons to human studies discussed here relate to IS. Although there are extensive genetic and physiological similarities between teleosts and mammals, anatomical differences make direct biomechanical comparisons difficult. For example, the teleost spine is not weight bearing due to the buoyancy from the swim bladder and the density of water [52]. That most human spinal curvatures are manifest in the coronal plane and the *curveback *deformity is in the sagittal plane may reflect the different muscle and ligament organization and consequently different tensions on the spine. Indeed, in response to pinealectomy, chickens demonstrate 3-dimensional spinal curvature with vertebral wedging [53], but salmon demonstrate spinal curvature in the sagittal plane only [52].

## Conclusions

With regard to idiopathic-type spinal curvature, the relationship between predisposing factors, growth, and mechanical stress is unknown. Although the *curveback *guppy is an appropriate model for understanding the basic biological systems involved in idiopathic-type curvature, its use for biomechanical study that directly relates to humans is limited. However, once molecular mechanisms involved in idiopathic-type spinal curvature are defined, it will be important to understand them in the context of growth and biomechanics, and therefore exploration of structural aspects of *curveback *curvature is important.

Based on similarities to human spinal curvatures and to animals with induced curves, the concave-convex biases described in the guppy suggest that the progression of curvature during growth is biomechanically mediated in *curveback*. However, this study does not allow us to discern whether the vertebral anomalies described in *curveback *are primary or secondary to the curve. Further studies with *in-vivo *imaging technology may allow for a better understanding of how vertebral shape changes relate to curvature during growth. Further experimentation should also investigate the sexual dimorphisms reported in this study in order to test for an association with severe curves among females in the *curveback *population.

## Competing interests

The authors declare that they have no competing interests.

## Authors' contributions

KFG conceived of the study, participated in the design of the study, performed the statistical analysis, and drafted and revised the manuscript. GRH carried out the histological analysis, participated in the design and final draft of the manuscript. JG carried out whole mount staining and measurements taken on vertebrae. RW carried out whole mount staining. FB participated in the design and final draft of the manuscript. All authors have read and approved the final manuscript.
